# Supplementary material for: Characterization and Homology Modeling of Catalytically Active Recombinant PhaCAp Protein from Arthrospira platensis
Source: Biology (Basel). 2023 May 20;12(5):751. doi: 10.3390/biology12050751 (PMC10215190; doi:10.3390/biology12050751)
Supplement: Supplementary file 1 [file biology-12-00751-s001.zip › biology-2354239-supplementary.pdf]

Supplementary Materials for
"Characterization and Homology Modeling of Catalytically Active Recombinant PhaC$_{Ap}$ Protein from *Arthrospira platensis*"

Chanchanok Duangsri [1], Tiina A. Salminen [2], Marion Alix [2], Sarawan Kaewmongkol [1], Nattaphong Akrimajirachoote [3], Wanthanee Khetkorn [4], Sathaporn Jittapalapong [1], Pirkko Mäenpää [5], Aran Incharoensakdi [6,7], Wuttinun Raksajit [1,*]

[1] Program of Animal Health Technology, Faculty of Veterinary Technology, Kasetsart University, Bangkok 10900, Thailand; fvetspj@ku.ac.th (S.J.)
[2] Structural Bioinformatics Laboratory and InFLAMES Research Flagship Center, Biochemistry, Faculty of Science and Engineering, Åbo Akademi University, 20520 Turku, Finland
[3] Department of Physiology, Faculty of Veterinary Medicine, Kasetsart University, Bangkok 10900, Thailand
[4] Division of Biology, Faculty of Science and Technology, Rajamangala University of Technology Thanyaburi (RMUTT), Thanyaburi, Pathumthani 12110, Thailand
[5] Faculty of Technology, University of Turku, 20014 Turku, Finland
[6] Laboratory of Cyanobacterial Biotechnology, Department of Biochemistry, Faculty of Science, Chulalongkorn University, Bangkok 10330, Thailand
[7] Academy of Science, Royal Society of Thailand, Bangkok 10300, Thailand
* Correspondence: cvtwnr@ku.ac.th

This file includes:

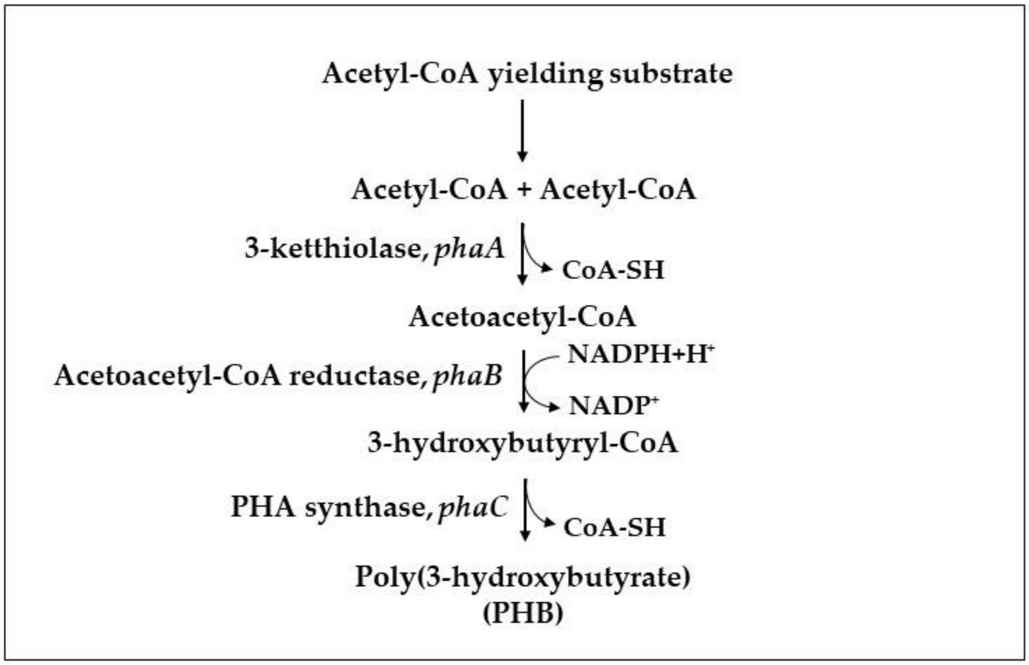

Figure S1. PHB Biosynthetic pathway in cyanobacteria (modified from Singh et al. 2017)
